# Multivariate characterization of white matter heterogeneity in autism spectrum disorder

**DOI:** 10.1016/j.nicl.2017.01.002

**Published:** 2017-01-06

**Authors:** D.C. Dean, N. Lange, B.G. Travers, M.B. Prigge, N. Matsunami, K.A. Kellett, A. Freeman, K.L. Kane, N. Adluru, D.P.M. Tromp, D.J. Destiche, D. Samsin, B.A. Zielinski, P.T. Fletcher, J.S. Anderson, A.L. Froehlich, M.F. Leppert, E.D. Bigler, J.E. Lainhart, A.L. Alexander

**Affiliations:** a Waisman Center, University of Wisconsin-Madison, Madison, WI, USA; b Department of Psychiatry, Harvard School of Medicine, Boston, MA, USA; c Child and Adolescent Psychiatry, McLean Hospital, Belmont, MA, USA; d Occupational Therapy Program, Department of Kinesiology, University of Wisconsin-Madison, Madison, WI, USA; e Department of Radiology, University of Utah, Salt Lake City, UT, USA; f Department of Pediatrics, University of Utah and Primary Children's Medical Center, Salt Lake City, UT, USA; g Department of Human Genetics, University of Utah, Salt Lake City, UT, USA; h Department of Psychology, University of Wisconsin-Madison, Madison, WI, USA; i Department of Psychiatry, University of Wisconsin-Madison, Madison, WI, USA; j Department of Neurology, University of Utah, Salt Lake City, UT, USA; k Scientific Computing and Imaging Institute, University of Utah, Salt Lake City, UT, USA; l School of Computing, University of Utah, Salt Lake City, UT, USA; m Interdepartmental Program in Neuroscience, University of Utah, Salt Lake City, UT, USA; n Department of Psychology, Brigham Young University, Provo, UT, USA; o Neuroscience Center, Brigham Young University, Provo, UT 84602, USA; p Department of Medical Physics, University of Wisconsin-Madison, Madison, WI, USA

**Keywords:** Autism spectrum disorder, Mahalanobis distance, Brain variability, Diffusion tensor imaging, White matter microstructure

## Abstract

The complexity and heterogeneity of neuroimaging findings in individuals with autism spectrum disorder has suggested that many of the underlying alterations are subtle and involve many brain regions and networks. The ability to account for multivariate brain features and identify neuroimaging measures that can be used to characterize individual variation have thus become increasingly important for interpreting and understanding the neurobiological mechanisms of autism. In the present study, we utilize the Mahalanobis distance, a multidimensional counterpart of the Euclidean distance, as an informative index to characterize individual brain variation and deviation in autism. Longitudinal diffusion tensor imaging data from 149 participants (92 diagnosed with autism spectrum disorder and 57 typically developing controls) between 3.1 and 36.83 years of age were acquired over a roughly 10-year period and used to construct the Mahalanobis distance from regional measures of white matter microstructure. Mahalanobis distances were significantly greater and more variable in the autistic individuals as compared to control participants, demonstrating increased atypicalities and variation in the group of individuals diagnosed with autism spectrum disorder. Distributions of multivariate measures were also found to provide greater discrimination and more sensitive delineation between autistic and typically developing individuals than conventional univariate measures, while also being significantly associated with observed traits of the autism group. These results help substantiate autism as a truly heterogeneous neurodevelopmental disorder, while also suggesting that collectively considering neuroimaging measures from multiple brain regions provides improved insight into the diversity of brain measures in autism that is not observed when considering the same regions separately. Distinguishing multidimensional brain relationships may thus be informative for identifying neuroimaging-based phenotypes, as well as help elucidate underlying neural mechanisms of brain variation in autism spectrum disorders.

## Highlights

• Mahalanobis distance proposed to characterize brain differences from a multivariate set of neuroimaging measures.
• Brain variation in autism is examined using Mahalanobis distance is sensitive to underlying microstructure.
• Comparison of Mahalanobis distance with traditional univariate measures is examined.
• Utility of multivariate brain relations with Mahalanobis distance and prospect of future studies are described.

## 1 Introduction

Neuroimaging has played an important role in understanding the neurobiological basis of many neurodevelopmental and psychiatric disorders. The majority of these studies have examined brain differences at a group level, often comparing a population of interest to a healthy or psychiatric control population. These studies are informative for identifying patterns of brain differences associated with a particular group or disorder; however, the assumption that the pathology of these disorders are consistent across all individuals with the disorder is inherently implied and implausible (Kim et al., 2013). While general morphological, organizational, and functional features of the brain are conserved during typical development within our species, and consistency of brain differences between individuals with developmental neuropsychiatric disorders and other groups may exist, the human brain is remarkably variable across individuals (Frost and Goebel, 2012, Kennedy et al., 1998, Lange et al., 1997, Mueller et al., 2013, Rademacher et al., 2001, Zilles and Amunts, 2013), suggesting that patterns of individual brain differences may help elucidate the neural mechanisms involved. This may be especially likely in clinically multifaceted, etiologically heterogeneous neurodevelopmental disorders, such as autism spectrum disorder (ASD), which emerges and is sustained in the midst of a complex cascade of interacting biological events and life experiences.

ASD results from atypical brain development and is clinically recognized by clustering, within affected individuals, of behaviors indicating abnormal development of reciprocal social interaction and social communication and unusual patterns of highly restricted and repetitive behaviors and interests (American Psychiatric Association, 2013). The term “spectrum” reflects that it is not yet possible to reliably distinguish or validate clinically meaningful subcategories of ASD at the clinical, biomarker, or neuroimaging level. In addition, ASD presents with a wide range of diverse symptom that may vary in severity (Geschwind and Levitt, 2007), frequent association with a variety of co-occurring neuropsychiatric and medical conditions, some symptom overlap with other disorders (Hutton et al., 2008, Tuchman, 2015, Zafeiriou et al., 2007) and variable (but typically early) age of onset across diagnosed individuals (Ozonoff et al., 2008, Werner and Dawson, 2005). Though the prevalence of the disorder has recently been estimated to affect 1 in 68 children (CDC, 2014), some children begin to show delays within the first 12 months of life, while others (25%–40%) regress from typical development to autism-like behaviors between 12 and 24 months (Werner and Dawson, 2005). Moreover, individual phenotypes of ASD are likely influenced by complex gene-environment interactions. For instance, many genes of small effect are heritable and contribute to the observable characteristics of ASD, while these characteristics are further modulated by one's environment via epigenetic mechanisms (Abrahams and Geschwind, 2008, Jeste and Geschwind, 2014).

The heterogeneity of the autism phenotype has similarly made the interpretation of brain imaging studies examining the neurobiological basis of ASD challenging. Magnetic resonance imaging (MRI) studies have suggested that the pathogenesis of autism involves alterations to brain volume (Courchesne et al., 2001, Hazlett et al., 2005, McAlonan et al., 2005, Piven et al., 1995), as well as widespread region-specific differences across the brain, involving the cerebellum, amygdala, and thalamus (Hardan et al., 2006, Hazlett et al., 2005, Nacewicz et al., 2006, Schumann et al., 2004), among others (for review, see (Amaral et al., 2008, Anagnostou and Taylor, 2011, Ecker and Murphy, 2014, Lainhart, 2006, Verhoeven et al., 2010)). Similarly, diffusion tensor imaging (DTI) studies have further revealed differences across white matter microstructure (for review, see (Travers et al., 2012)), while other imaging strategies have associated ASD with alterations of cortical thickness (Hazlett et al., 2011, Wallace et al., 2015, Zielinski et al., 2014) and brain connectivity (Anderson et al., 2011, Belmonte et al., 2004, Just et al., 2012, Kleinhans et al., 2008, Vissers et al., 2012). Furthermore, group differences have been observed to be dynamic, as recent studies have highlighted disparities of developmental trajectories between individuals with and without autism (Hazlett et al., 2011, Lange et al., 2015, Travers et al., 2015b, Wolff et al., 2015, Wolff et al., 2012, Zielinski et al., 2014).

Despite a growing body of literature showing significant group differences across an assortment of neuroimaging techniques, there has been poor replication across studies of autism, while many of these studies report greater neuroanatomical variability across the ASD group (Amaral et al., 2008, Anagnostou and Taylor, 2011, Byrge et al., 2015, Hahamy et al., 2015, Hasson et al., 2009, Minshew and Keller, 2010, Travers et al., 2012). While this variability may stem from the variation of study populations or different analytical techniques, it is clear from the range of findings that the neuroanatomy of ASD, as its behavioral counterpart, is not restricted to a single brain region, specific network or even a common biological mechanism. Instead, these differences appear to be subtle and widespread (Ecker et al., 2010), and these differences potentially vary from person-to-person. Being able to characterize each person with ASD based on the degree of how the multidimensional measures of his/her brain differ from the general population would provide a method to numerically represent the degree of brain abnormality in each individual. Thus, there is a critical need to better characterize the distributions of these previous measures on the individual level in order to advance the clinical utility of brain imaging findings in autism.

The extent to which multivariate brain relationships may enable better characterization and discrimination at the individual level, as compared to commonly utilized univariate measures, remains unclear. Distinguishing such relationships, however, is critical as a multivariate description could be informative for identifying phenotypical subgroups, better understanding individual brain changes, and guiding personalized therapies or interventions (Brammer, 2009, Kumar, 2011, Li, 2011). Multivariate analysis techniques, which seek to evaluate multiple measures simultaneously, have great potential to discern underlying relationships in a wide variety of neuroimaging applications, including assessment of clinical outcomes and diagnosis, characterizing disease etiology and progression, as well as evaluating neurodevelopmental and neurodegenerative diseases (Habeck et al., 2010, Levman and Takahashi, 2015, McIntosh and Mišić, 2013). While a wide variety of multivariate strategies exist, one such multivariate measure which is an extension of the Euclidean distance, known as the Mahalanobis distance (D_M_; (Mahalanobis, 1936)), may be informative for distinguishing such multivariate brain relationships. D_M_ has been applied and shown to be informative in various neuroimaging contexts, including signal outlier detection (Fritsch et al., 2012), differentiating brain tissue types (Taxt and Lundervold, 1994), classifying neurological diseases (Caprihan et al., 2008, Lindemer et al., 2015), and evaluating relationships of development (Kulikova et al., 2014) and connectivity (Shehzad et al., 2014). Hence, D_M_ may be advantageous to detect individual brain differences in neurodevelopmental disorders, such as ASD, however, this remains unapproached.

In this study, we utilize D_M_ to characterize individual brain differences in a large, longitudinal sample of individuals with and without ASD. Specifically, we describe a framework which to measure D_M_ from this longitudinal cohort and investigate whether multivariate white matter microstructural features distinguish individuals with ASD from their typically developing peers. We further examine the degree to which microstructural characteristics are associated with measures of autism symptom severity.

## 2 Materials and methods

### 2.1 Participants

**Fig. 1:**
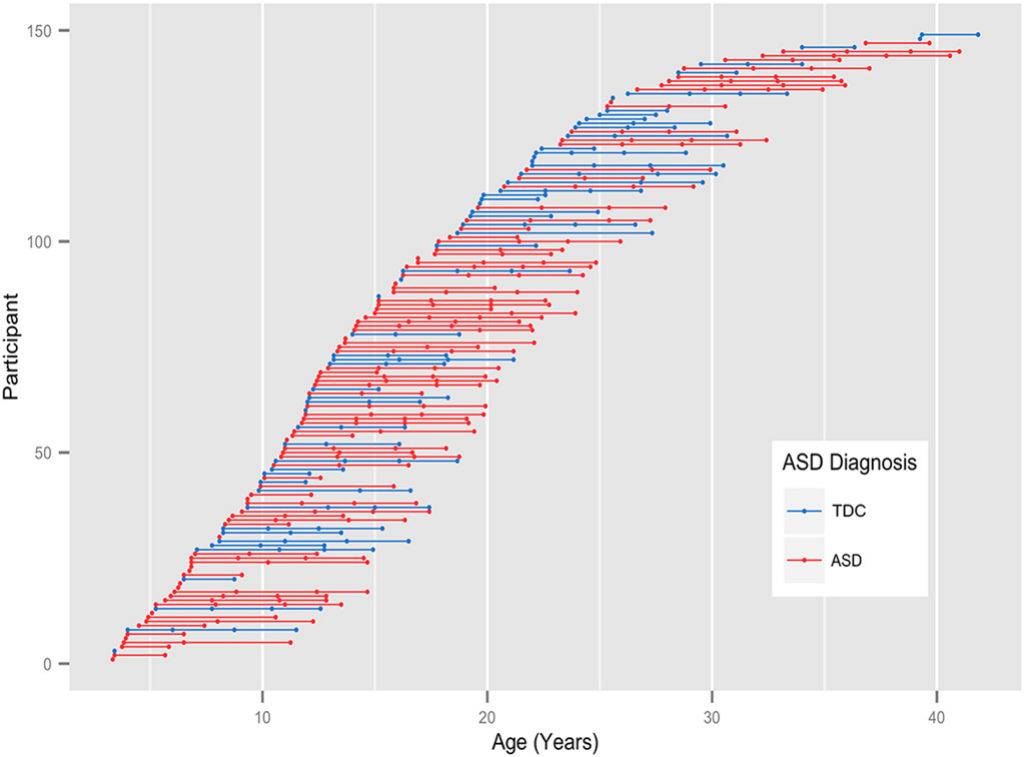
Longitudinal sampling of ASD and TDC participants. Ages of the 149 subjects at each scan. The age of each subject at each time point is denoted along each row and the repeated measurements are connected with a solid line. Individuals diagnosed with ASD (red) and TDC (blue) are additionally distinguished. (For interpretation of the references to color in this figure legend, the reader is referred to the web version of this article.)

The institutional review boards of the University of Utah and University of Wisconsin-Madison approved the study protocol and procedures. Participants consisted of 92 individuals with ASD and 57 typically developing controls (TDC), selected from a broader longitudinal study of brain development in autism and typical development (Lange et al., 2015, Travers et al., 2015b, Travers et al., 2015a, Travers et al., 2014, Zielinski et al., 2014). Consent was obtained from all adult participants, and both parental consent and participant assent were obtained for participants under the age of 18 years. Exclusion criteria consisted of: history of severe head injury, seizure disorder, hypoxia-ischemia, genetic disorder associated with ASD (identified with Fragile-X testing or karyotype), known medical cause of ASD diagnosis (e.g. known patient history, and physical exam), and/or other neurological disorders. All participants were male and ranged in age from 3.3 and 36.8 years at the time of enrollment. Participants were recruited and clinically assessed; they underwent MRI scanning one to four times as part of an accelerated longitudinal study design over an approximately 10-year period (Harezlak et al., 2005; see Fig. 1). Fifty-seven participants had four scans (42 ASD and 15 TDC), 32 participants had three scans (18 ASD and 14 TDC), 37 participants had two scans (18 ASD and 19 TDC), and 23 participants had one scan (14 ASD and 9 TDC). The average interval between scans was 2.6 years. See Table 1 for additional participant information.

**Table 1:** Demographic characteristics of the longitudinal ASD and TDC groups.

	ASD	TDC	p-Value
Number of subjects	92	57	–
Mean age (years)	18.15 (8.47)	19.16 (7.95)	0.23
Mean scans per subject (Std. dev)	2.96 (1.13)	2.61 (1.05)	0.06
Mean inter-scan interval (Std. dev) [years]	2.82 (0.83)	2.77 (1.02)	0.69
Total number of scans	272	149	–
Number of scans at time 1	92	57	–
Number of scans at time 2	78	48	–
Number of scans at time 3	60	29	–
Number of scans at time 4	42	15	–
Mean FSIQ (Std. dev)	99.42 (17.70)	118.17 (13.23)	< 0.001
Mean PIQ (Std. dev)	102.24 (18.10)	116.34 (14.76)	< 0.001
Mean VIQ (Std. dev)	95.06 (21.63)	114.72 (13.21)	< 0.001
Mean total raw SRS (Std. dev)	99.73 (30.30)	15.57 (11.79)	< 0.001

Participants with ASD were diagnosed according to the Autism Diagnostic Interview-Revised (ADI-R; (Lord et al., 1994)), Autism Diagnostic Observation Schedule-Generic (ADOS-G; (Lord et al., 2000)), Diagnostic Statistical Manual-IV (American Psychiatric Association, 1994), and the International Statistical Classification of Diseases and Related Health Problems-10th revision (ICD-10) criteria (World Health Organization, 2007). Typical development was confirmed by performing standardized psychiatric assessment, neuropsychological assessment, IQ testing and assessment with the ADOS-G (Lord et al., 2000). All ASD and TDC participants received IQ testing at each time point, providing indices of verbal (VIQ), performance (PIQ) and full-scale (FSIQ) IQ. The Social Responsiveness Scale (SRS; Constantino, 2002), a standardized parent-report questionnaire that assesses the degree of ASD symptom severity, was additionally completed; however, as the age range of the present study extended beyond the normed age range for the SRS (4–18 years), total raw scores are reported. Individuals with less socially reciprocal behavior are indicated by higher total raw SRS scores.

### 2.2 Imaging protocol

A total of 421 scans were acquired from the participants (272 ASD, 149 TDC). All magnetic resonance images were collected on a Siemens Tim Trio 3.0 T scanner at the University of Utah. At each time point, diffusion-weighted imaging (DWI) data were obtained using a single shot spin-echo echo-planar imaging pulse sequence. Bipolar gradients with dual-echo refocusing was used to reduce eddy currents (Reese et al., 2003), while parallel acquisition, with a geometric reduction factor of two, was used to reduce image distortions from magnetic field inhomogeneities and acquisition time. Imaging parameters consisted of: repetition time: 7000 ms; echo time: 84 ms at time 1, and 91 ms at times 2, 3, 4; and bandwidth: 1346 Hz/pixel. A 25.6 cm × 25.6 cm imaging field of view was used in conjunction with an acquisition matrix of 128 × 128 to provide a 2 mm × 2 mm in-plane resolution. Coverage across the cerebrum and cerebellum was achieved by acquiring 60 axial-oriented contiguous slices with a slice thickness of 2.5 mm. Diffusion data were acquired with diffusion encoded along 12 non-collinear directions with b = 1000s/mm^2^ and a single non-diffusion weighted (b = 0 s/mm^2^) image. The acquisition was averaged across four repeats for a total imaging time of 6.5 min.

To improve prospects of successful DTI acquisition, participants were able to practice lying in a mock MRI scanner. Sedation was offered to young participants with ASD, using a combination of remifentanil and propofol, if needed to improve data quality. In total, 47 of the 434 longitudinal scans were acquired under a strict clinical sedation protocol approved by the University of Utah's Institutional Review Board and that was performed and monitored by an onsite faculty anesthesiologist. A total motion index (TMI) was recorded to account for the effects of potential group differences in head motion during MRI scanning (Benner et al., 2011, Yendiki et al., 2014) and used in later analyses. Between times 1 and 2, the scanner hardware and software underwent an upgrade, resulting in a head coil change (8-channel receive-only coil at time 1 and a 12-channel receive-only coil at times 2, 3, 4) and alteration of the DWI echo time (described above), however, there was no significant group difference of the proportion of scans prior to and following the scanner upgrade (Z = − 0.51; p = 0.61).

### 2.3 Image analysis

All image processing and analyses were conducted at the University of Wisconsin-Madison and were processed using methods previously described (Travers et al., 2015b). Individual diffusion weighted images were co-registered to account for any subtle distortion, translation and rotation from bulk head motion and eddy currents using an affine registration tool (Jenkinson et al., 2002) from the FMRIB software library (FSL; http://fsl.fmrib.ox.ac.uk/fsl/fslwiki/) suite, while gradient directions were corrected for rotations (Leemans and Jones, 2009). FSL's brain extraction tool (BET; (Smith, 2002)) was used to remove non-parenchyma signals. Diffusion tensors were fit at each voxel using the robust estimation of tensors by outlier rejection (RESTORE; (Chang et al., 2005)) algorithm as part of the Camino software package (Cook et al., 2006). Eigenvalues (λ_1_, λ_2_, λ_3_) were calculated from these voxel-wise estimates of the diffusion tensor and quantitative maps of fractional anisotropy (FA – normalized standard deviation of the eigenvalues reflecting the relative degree of diffusion anisotropy), mean diffusivity (MD – average of the eigenvalues), axial diffusivity (AD – largest eigenvalue), and radial diffusivity (RD – third eigenvalue corresponding to diffusion perpendicular to the major eigenvector were derived (Basser and Pierpaoli, 1996). Quantitative maps were visually inspected for artifacts (i.e. slice intensity banding, FA hyper-intensities, distortions, and/or blurring).

To reduce subtle alignment inconsistencies that might result from not accounting for the repeated measurements (Aubert-Broche et al., 2013, Dean et al., 2014b), a longitudinal registration pipeline was developed to align individual DTI measurements to a common template space. For each participant, an initial diffusion tensor template was created from all the acquired longitudinal time points using affine and diffeomorphic diffusion tensor registration, as implemented in DTI-TK (Zhang et al., 2006). DTI-TK was then used to generate an overall, un-biased population-specific template from these subject-specific diffusion tensor templates. White matter tracts from the JHU ICBM-DTI-81 template (Mori et al., 2005, Oishi et al., 2008) were spatially aligned to this population template using the Advanced Normalization Tools diffeomorphic spatial registration (Avants et al., 2008) and nearest neighbor interpolation. The normalized JHU ICBM-DTI-81 template was warped into each subject's native space by applying the inverse of the spatial transformations estimated in the population- and subject-specific template generation step. A subset of 11 regions of interest (ROIs) reported to be implicated in ASD (Travers et al., 2012) were selected from the available 48 labels contained within the JHU template for subsequent analyses. These regions included: genu, body, and splenium of the corpus callosum; superior longitudinal fasciculus, internal capsules (anterior and posterior portions); corticospinal tract; uncinate fasciculus; cingulum; superior fronto-occipital fasciculus; and sagittal stratum (i.e. inferior longitudinal fasciculus and inferior fronto-occipital fasciculus). Left and right homologous pairs were additionally used where appropriate, for a total of 19 ROIs examined. For each longitudinal time point and anatomical region, the median FA, MD, AD, and RD values were extracted from each participant's corresponding native-space FA MD, AD, and RD maps, as the median is less sensitive to voxels with extreme values (van Belle et al., 2004).

### 2.4 Calculating the Mahalanobis distance

The Mahalanobis distance (D_M_; (Mahalanobis, 1936)) is a multivariate extension of the Euclidean distance, measuring the distance of each member of a set of multivariate measures to the mean of their multivariate distribution. For each subject, D_M_ is calculated using the following formula:

(1) $$D_{M}=\sqrt{\left(\vec{x}-\mu \right)S^{-1}{\left(\vec{x}-\mu \right)}^{T}}$$

where $\vec{x}$ corresponds to the set of multivariate neuroimaging observations for each individual, *μ* is the mean of the multivariate distribution of neuroimaging measures, and *S* is the variance-covariance matrix between measures. For example, if *m* measures are collected from each individual, $\vec{x}$ and *μ* correspond to a 1 × *m* vector, *S* corresponds to an *m* × *m* matrix, and D_M_ from Eq. (1) is a scalar. In this way, D_M_ accounts for the variance of individual observations as well as the covariance between the set of observations, homologous to the Euclidean distance in univariate analysis. In the present study, we estimate D_M_ for individuals with ASD from their corresponding DTI brain measures, using the TDC as the population reference. In this case, D_M_ corresponds to how close an ASD individual's brain measures are to the multivariate mean of the TDC population, where larger D_M_ represents increased distance from the center of the typically developing population.

In constructing D_M_ from longitudinal measurements, it is important to account for neurodevelopmental processes (Dean et al., 2014b, Dean et al., 2014a, Lebel and Beaulieu, 2011, Lebel et al., 2012, Snook et al., 2005). Generalized additive mixed models (GAMM's) were fit to the regional developmental trajectories of the DTI parameters (FA, MD, AD, and RD) of the TDC group to characterize the observed age-related white matter changes and establish a normative growth trajectory for each brain region. Generalized additive mixed models were utilized to characterize the age-related changes as these models have been designed specifically for cohort-sequential longitudinal designs (Wood, 2006, Wood, 2012) and for their ability to account for repeated measurements from the same individual. Furthermore, since the growth model that describes white matter is unknown, the semi-parametric nature of these spline models provides flexibility in capturing subtle developmental changes compared to parametric growth models (Travers et al., 2015b). Longitudinal modeling analyses were performed using R version 3.2.1 (R Development Team, 2014), while accounting for the nuisance variables of head coil (due to upgrade discussed earlier) and total motion index.

Upon determining the best fit model of the TDC group, these models were used to predict FA, MD, AD and RD along the modeled TDC growth trajectory for every ASD participant at each time point and for each brain region. The difference between the participants' parameter values from these predicted values (i.e. the model residuals) were calculated, corresponding to the vertical distance between the participant parameter measurements and the TDC reference growth trajectory. D_M_ for each time point was calculated from these residuals using eq. (1), where $\vec{x}-\mu$ corresponds to the difference between observed measurements ($\vec{x}$) and modeled values (*μ*), and *S* is the variance-covariance matrix of the modeled residuals from the TDC group.

D_M_ was similarly calculated for each TDC individual. However, to avoid including an individual's measurements in the model fitting when establishing the reference growth trajectory, a leave-one-out approach was used when modeling regional FA, MD, AD and RD developmental trajectories. For a given TDC subject, FA, MD, AD, and RD longitudinal measurements were removed prior to modeling and participant-specific residuals and D_M_ was calculated as before. This process was repeated for each TDC participant.

**Supplementary Fig. 1:**
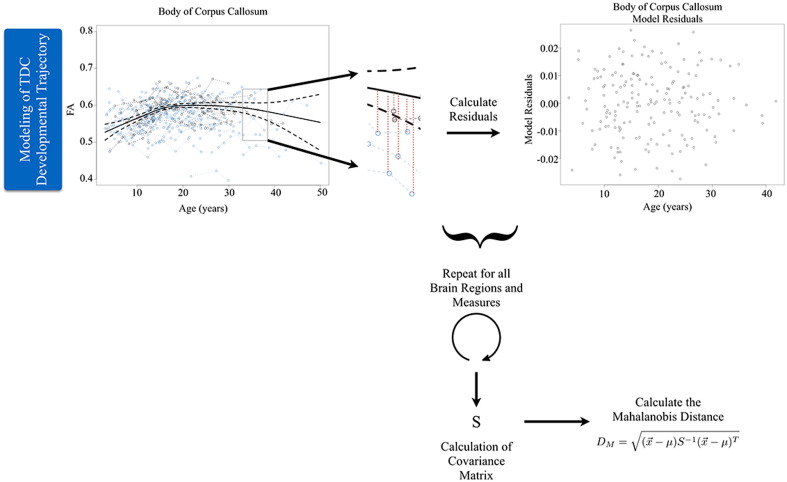
Illustrative schematic of Mahalanobis distance (DM) calculation. First, the normative models of each brain region are constructed from the neuroimaging data of the typically developing controls. The model residuals from these models are used to construct the covariance matrix, S. Using these normative models, the difference between the model and observed neuroimaging measure for each brain region is calculated and used to compute DM.

After calculating D_M_ for each longitudinal time point, an average across these time points was computed for each participant, providing a single, representative D_M_ value for each individual. Supplementary Fig. 1 displays a representative schematic illustrating the process of calculating D_M_. Distributions of Mahalanobis distances were generated for both the ASD and TDC groups and the Bhattacharyya coefficient (Mo et al., 2015) was computed to assess the degree of overlap between the group distributions, where smaller Bhattacharyya coefficients correspond to a lesser degree of overlap. D_M_ was additionally calculated by considering regional DTI parameters (FA, MD, AD, and RD) separately.

### 2.5 Construction of univariate DTI distributions

Standard scores were similarly calculated for each ROI and DTI parameter (i.e. FA, MD, AD, RD). Longitudinal mixed effects models were used to characterize the age-related changes of the TDC group and establish a normative reference growth curve. Model residuals were calculated and normalized by the standard deviation of fit residuals. Standard scores for TDC participants were again calculated using a leave-one-out approach. Individual time points were aggregated by calculating the mean of individual time point standard scores, producing a single standard score for each individual. Distributions of regional standard scores were generated and again the Bhattacharyya coefficient was computed to measure distributional overlap.

## 3 Results

**Fig. 2:**
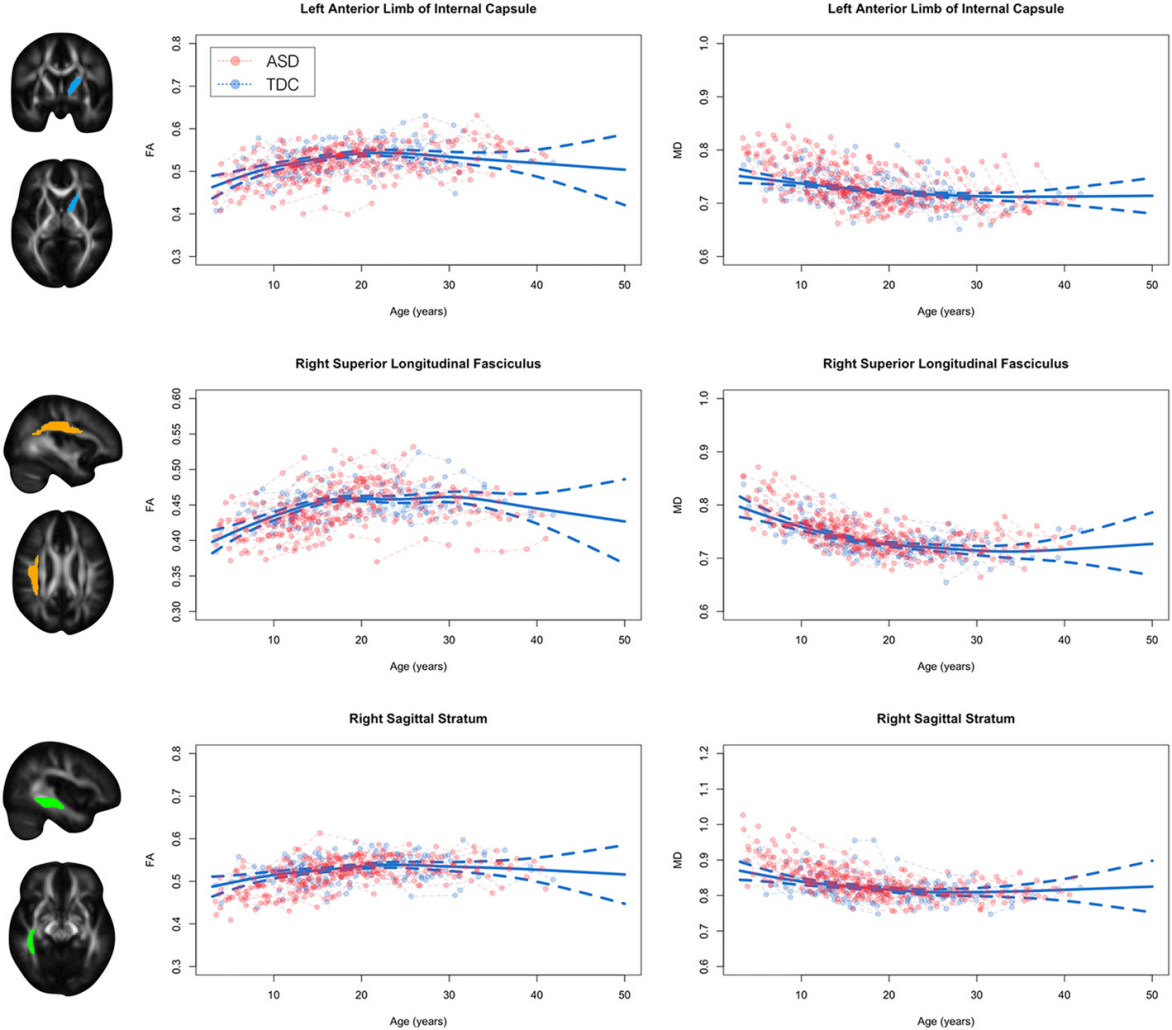
Developmental trajectories of white matter in ASD and TDC participants. Representative developmental trajectories of FA and MD from left hemispheric anterior limb of the internal capsule (*top*), right hemispheric superior longitudinal fasciculus (*middle*), and right hemispheric sagittal stratum (*bottom*). ASD (red) and TDC (blue) longitudinal observations are denoted by circles while dashed lines connect repeated time points for specific subjects. The solid blue line corresponds to the best fit generalized additive mixed-effects model, corresponding to the normative reference growth trajectory. (For interpretation of the references to color in this figure legend, the reader is referred to the web version of this article.)

**Supplementary Fig. 2:**
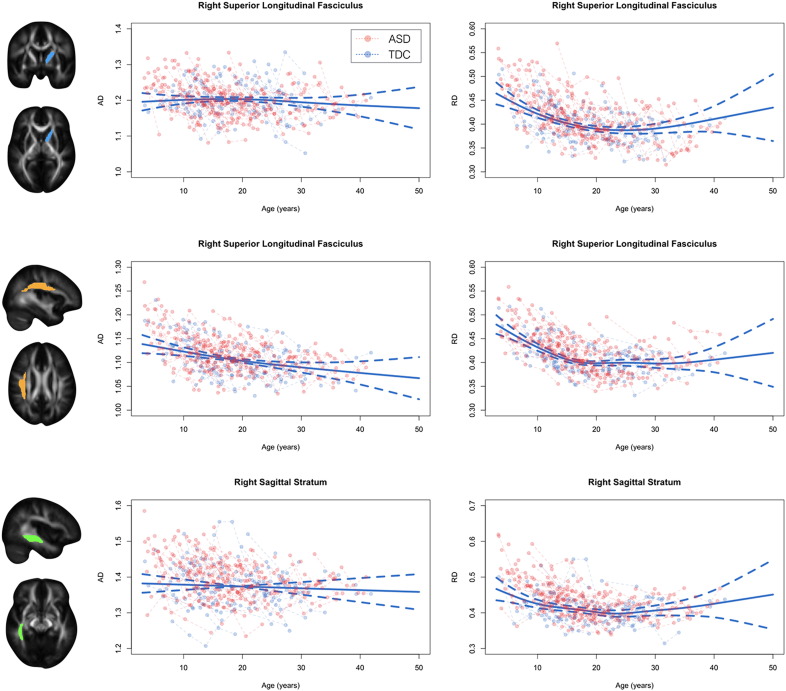
Developmental trajectories of white matter in ASD and TDC participants. Representative developmental trajectories of AD and RD from left hemispheric anterior limb of the internal capsule (*top*), right hemispheric superior longitudinal fasciculus (*middle*), and right hemispheric sagittal stratum (*bottom*). ASD (blue) and TDC (black) longitudinal observations are denoted by circles while dashed lines connect repeated time points for specific subjects. Solid black line corresponds to the best fit generalized additive mixed-effects model, corresponding to the normative reference growth trajectory.

Representative FA and MD trajectories for the ASD and TDC group are displayed in Fig. 2 (AD and RD trajectories displayed in Supplemental Fig. 2). These trajectories highlight the significant age-related changes of DTI parameters that occur across the first four decades of life as well as visually depict neurodevelopmental differences between the ASD and TDC groups. It can be appreciated that the age-related changes are non-linear, with rapid changes (i.e. increases in FA and decreases in MD, AD, and RD) occurring at early ages and slower changes occurring into adulthood. To characterize the overall shape of the TDC growth curve and establish a population reference, generalized additive mixed-effects models were used. These models depict the complexity of the TDC growth trajectory by smoothly bending to the data at important nonlinear growth spurts or declines.

### 3.1 Mahalanobis distances

**Fig. 3:**
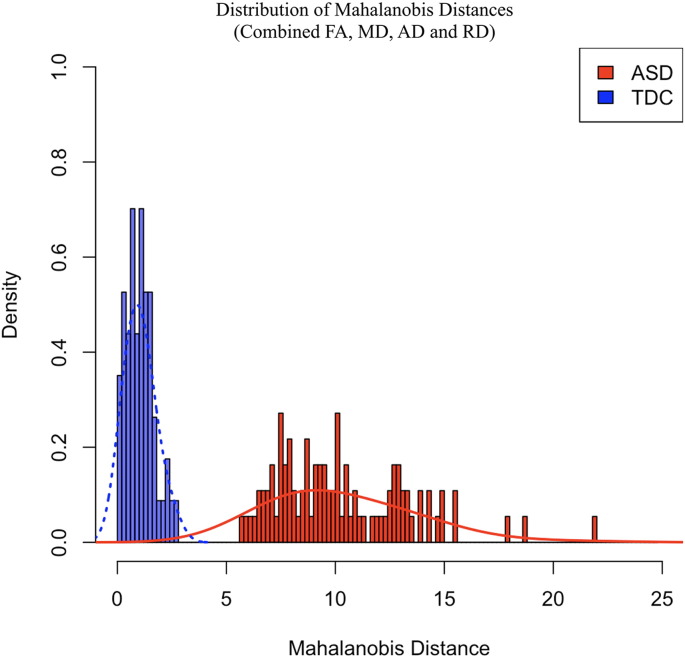
Mahalanobis distance distributions of white matter. ASD (red) and TDC (blue) distributions of Mahalanobis distance (D_M_) calculated from combined FA, MD, AD and RD measurements. Representative normal curves for each group are overlaid onto these distributions to illustrate the rightward shift and increased variability of D_M_ values in ASD subjects. (For interpretation of the references to color in this figure legend, the reader is referred to the web version of this article.)

Average D_M_ were computed for each ASD and TDC individual from the set of combined longitudinal regional brain FA, MD, AD, and RD measurements. The distribution of D_M_ across participants is shown in Fig. 3. It is evident that the ASD distribution of D_M_ values is shifted to the right and does not overlap with that of the TDC group, suggesting a larger mean and greater degree of microstructural deviation from the normative reference group. In comparing these group distributions, D_M_ values were found to be increased (*t(141)* = 27.911, p < 0.001) and more variable (*F(91,56)* = 23.17, p < 0.001) for the ASD individuals compared to the TDC participants. In particular, 92 of the 92 (100%) ASD participants had a D_M_ value greater than the TDC D_M_ mean (1.03), while 92 (%) had values greater than two standard deviations away from the TDC mean.

**Fig. 4:**
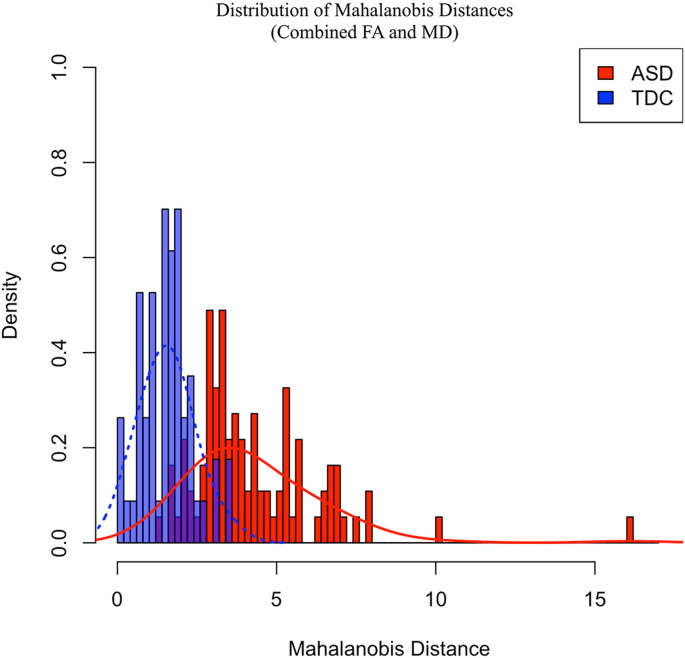
Combined FA and MD Mahalanobis distance distribution. ASD (red) and TDC (blue) distributions of Mahalanobis distance (D_M_) calculated from combined FA and MD measurements. Representative normal curves for each group are overlaid onto these distributions to illustrate the rightward shift and increased variability of D_M_ values in ASD subjects. (For interpretation of the references to color in this figure legend, the reader is referred to the web version of this article.)

We additionally calculated D_M_ from the set of longitudinal FA and MD measures (i.e. removing AD and RD), as these are most commonly reported DTI measures in the literature. The distribution of D_M_ calculated from FA and MD is shown in Fig. 4. Similar to the previous results, the ASD distribution of D_M_ values is shifted to the right compared to the TDC group, suggesting a larger mean and greater degree of microstructural deviation from the normative reference group. In comparing these group distributions, D_M_ values were found to be increased (*t(141)* = 11.28, p < 0.001) and more variable (*F(91,56)* = 6.91, p < 0.001) for the ASD individuals compared to the TDC participants. In this case, 91 of the 92 (98.9%) ASD participants had a D_M_ value greater than the TDC D_M_ mean (1.55), while 65 (70.7%) had values greater than two standard deviations away from the TDC mean.

**Fig. 5:**
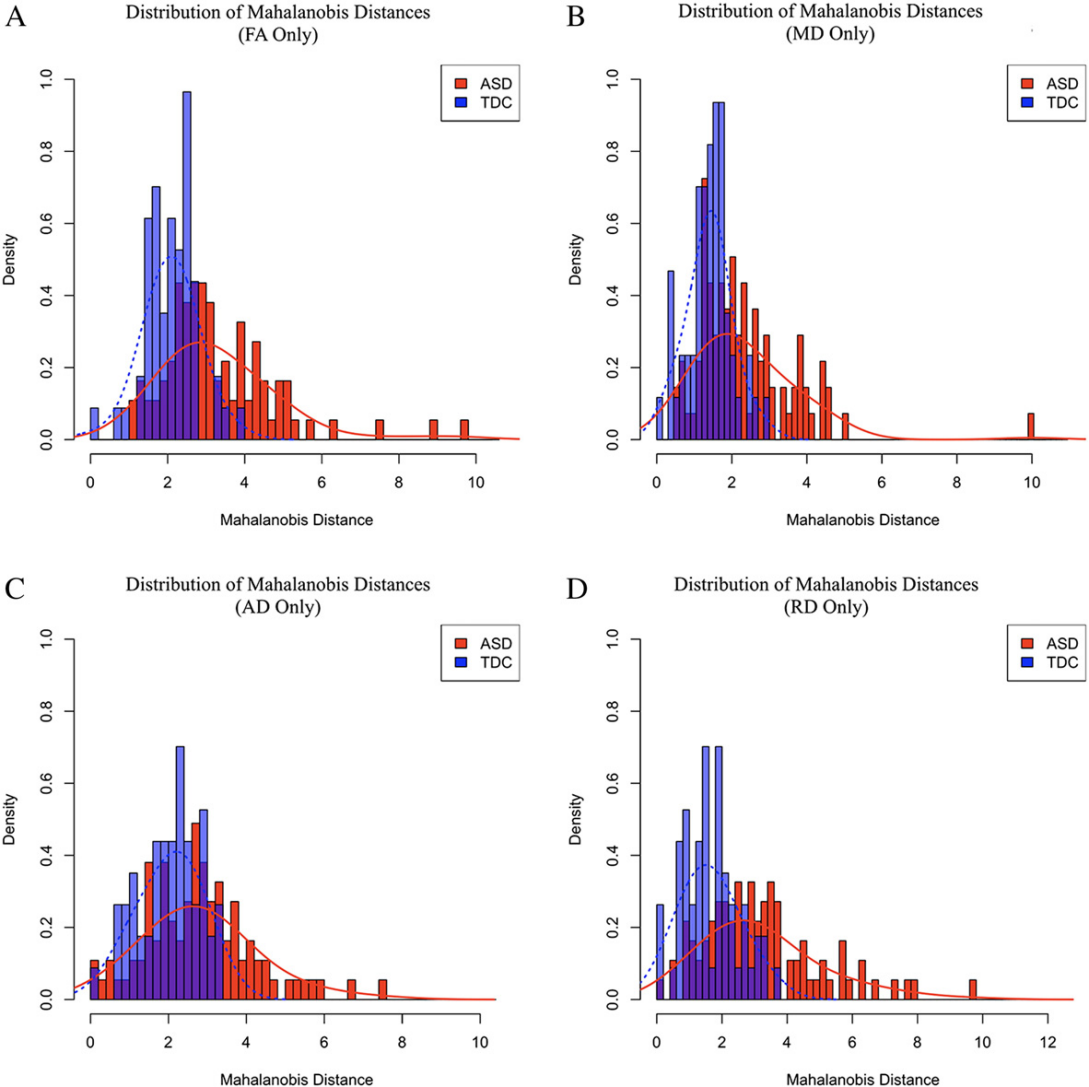
FA, MD, AD, and RD Mahalanobis distance distributions. Mahalanobis distance (D_M_) distributions calculated from (A) FA measurements only, (B) MD measurements only, (C) AD measurements only, and (D) RD measurements only. ASD individuals are shown in red while TDC participants are shown in blue. Representative normal curves for each group are overlaid onto these distributions to illustrate the rightward shift and increased variability of D_M_ values in ASD subjects. (For interpretation of the references to color in this figure legend, the reader is referred to the web version of this article.)

In addition, D_M_ was calculated separately for FA, MD, AD, and RD (Fig. 5a–d). Similar to D_M_ computed from combined DTI measurements, the distributions of D_M_ for each DTI parameter appear increased and more variable within the ASD group, suggesting each individual DTI parameter contributes to the microstructural deviations in ASD. In particular, the mean and standard deviations of the FA-, MD-, AD-, and RD-based ASD D_M_ distributions were found to be significantly different from the respective TDC distributions (FA-based D_M_: *t(141)* = 6.91, p < 0.001; *F(91,56)* = 5.16, p < 0.001; MD-based D_M_: *t(141)* = 6.01, p < 0.001; *F(91,56)* = 5.22, p < 0.001; AD-based D_M_: *t(141)* = 4.34, p < 0.001; *F(91,56)* = 3.30, p < 0.001; RD-based D_M_: *t(141)* = 6.01, p < 0.001; *F(91,56)* = 5.22, p < 0.001). With respect to the FA-based D_M_ values, 78 of 92 (84.8%) ASD individuals had scores greater than the TDC mean (2.08), and 35 of 92 (38.0%) had values greater than two standard deviations from the TDC mean. Similarly, 68 (73.9%) and 31 (33.7%) ASD individuals had MD-based D_M_ values greater than the TDC mean (1.81) and two standard deviations from the mean, respectively. For AD, 61 (66.3%) of ASD individuals had values greater than the TDC mean (2.02) and 22 (23.9%) had values greater than two standard deviations from the TDC mean, whereas 76 (82.6%) and 34 (40.9%) ASD individuals had RD-based D_M_ values greater than the TDC mean (1.63) and two standard deviations from the mean, respectively.

The distributions of D_M_ computed using all the DTI parameters between the ASD and TDC individuals were completely disjoint and therefore yielded a Bhattacharyya coefficient of zero. The next smallest Bhattacharyya coefficient was for the combined FA and MD coefficients (8.60), followed by RD-based D_M_ values (13.96), AD-based D_M_ values (15.81), FA-based D_M_ values (16.03), and finally the MD-based D_M_ values (16.09). This indicates that ASD and TDC distributions of D_M_ values have the greatest separation when calculated from each of the DTI parameters, suggesting each parameter is informative for assessing microstructural brain deviations of ASD.

### 3.2 Univariate DTI distributions

**Fig. 6:**
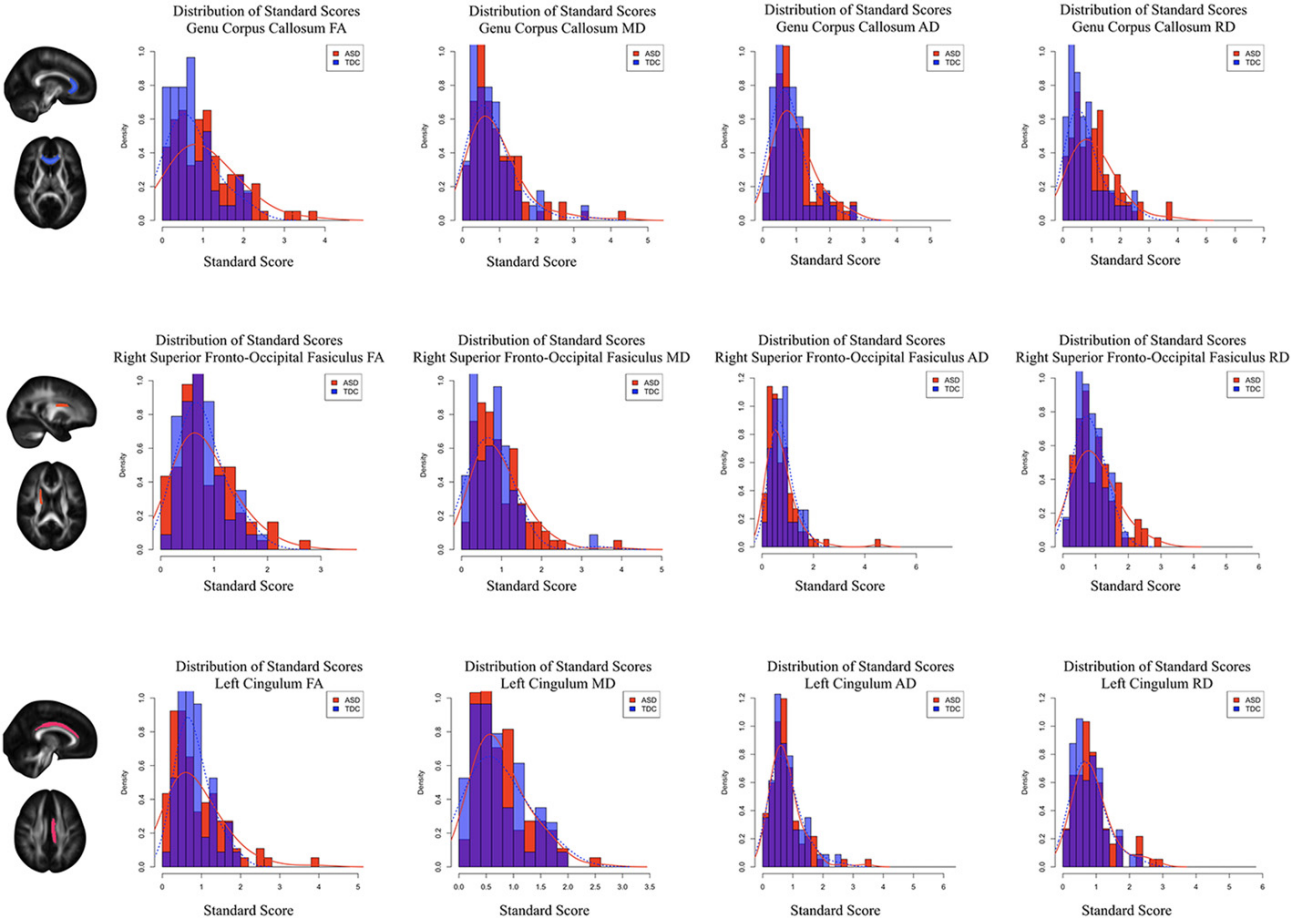
Standard score distributions of white matter. Representative FA, MD, AD, and RD distributions of univariate standard scores from the genu of the corpus callosum (*top*), right hemispheric superior fronto-occipital fasciculus (*middle*), and left hemispheric cingulum (*bottom*). Compared to distributions of D_M_, univariate distributions are less variable between ASD and TDC individuals.

We further compared the distribution of D_M_ values to univariate distributions of FA, MD, AD, and RD standard scores. Representative univariate distributions of the DTI parameters are shown in Fig. 6. To make these univariate measures comparable to D_M_ values, the absolute value of the standard score was taken. In most brain regions, standard scores are larger within the ASD group, suggesting larger FA, MD, AD, and RD deviations from the normative growth curve. Table 2 provides details for both the ASD and TDC group mean differences of these univariate distributions as well as whether the variability of these distributions differed. Of the examined brain regions, FA and RD of the body of the corpus callosum and AD and MD of the left posterior limb of the internal capsule had the largest group difference, while FA and RD in the genu of the corpus callosum, MD in the left superior fronto-occipital fasciculus, and AD in the left uncinate fasciculus had the least overlap between univariate standard score distributions. Comparing univariate Bhattacharyya coefficients to the Bhattacharyya coefficients derived from the D_M_ distributions highlights that D_M_ measures have less overlap (i.e. greater delineation) between ASD and TDC individuals than univariate-based measures.

**Table 2:** Comparison of standard score distributions between ASD and TDC groups. Differences between the group distribution means for each DTI parameter were assessed using t-statistics, while the differences in the variability of the distributions of DTI parameters were tested using F-statistics. Bhattacharyya coefficients were used to assess the overlap between ASD and TDC distributions.

Region	FA					MD					AD					RD				
	*t(141)*	p	*F(91,56)*	p	B. Coeff	*t(141)*	p	*F(91,56)*	p	B. Coeff	*t(141)*	p	*F(91,56)*	p	B. Coeff	*t(141)*	p	*F(91,56)*	p	B. Coeff
Genu of corpus callosum	3.10	< 0.001	1.91	0.01	22.47	1.05	0.30	1.40	0.17	25.36	1.92	0.06	1.37	0.20	25.42	2.45	0.02	1.43	0.15	22.29
Body of corpus callosum	3.97	< 0.001	2.94	< 0.001	22.74	2.60	0.01	3.23	< 0.001	25.02	0.27	0.78	1.03	0.93	26.40	3.81	< 0.001	3.67	< 0.001	22.78
Splenium of corpus callosum	2.21	0.03	2.86	< 0.001	23.66	2.22	0.03	2.41	< 0.001	25.20	− 0.19	0.85	0.98	0.92	26.81	3.48	< 0.001	3.94	< 0.001	22.58
Right anterior limb of internal capsule	1.00	0.32	1.54	0.08	25.92	0.26	0.79	1.29	0.30	26.83	0.65	0.52	1.15	0.58	25.79	3.38	< 0.001	1.30	0.29	25.26
Left anterior limb of internal capsule	0.65	0.52	1.14	0.60	26.55	1.39	0.17	1.16	0.56	24.00	0.61	0.54	0.84	0.45	27.33	1.07	0.28	1.29	0.31	25.24
Right posterior limb of internal capsule	0.87	0.38	0.60	0.03	25.75	2.60	0.01	1.38	0.20	26.59	− 0.75	0.46	1.35	0.22	26.87	2.20	0.03	1.43	0.15	25.79
Left posterior limb of internal capsule	− 0.54	0.59	0.57	0.02	26.19	2.75	0.01	1.79	0.02	26.72	0.34	0.74	1.03	0.93	25.85	1.01	0.32	1.22	0.42	27.59
Right superior longitudinal fasciculus	1.99	0.05	1.94	0.01	24.31	0.73	0.47	1.39	0.19	25.51	0.17	0.87	1.50	0.10	26.53	1.02	0.31	1.68	0.04	24.58
Left superior longitudinal fasciculus	1.50	0.14	1.62	0.05	24.43	0.84	0.40	1.37	0.20	25.98	− 0.54	0.59	1.03	0.92	26.55	1.81	0.07	1.88	0.01	24.78
Right corticospinal tract	− 0.90	0.37	0.97	0.88	27.89	− 0.89	0.38	1.39	0.18	27.82	0.07	0.94	0.65	0.07	27.64	− 0.94	0.35	0.92	0.72	28.86
Left corticospinal tract	1.17	0.24	1.39	0.18	25.02	− 0.50	0.62	1.47	0.12	28.11	− 2.43	0.02	0.64	0.06	26.66	0.82	0.41	0.85	0.49	26.59
Right uncinate fasciculus	0.42	0.68	1.14	0.60	26.68	2.72	0.01	0.97	0.87	27.17	1.68	0.10	1.05	0.87	26.94	1.71	0.09	1.11	0.69	26.80
Left uncinate fasciculus	0.31	0.75	1.07	0.79	26.12	0.40	0.69	1.01	0.97	26.42	1.48	0.14	0.77	0.26	24.49	− 0.47	0.64	0.59	0.03	25.85
Right cingulum	− 0.04	0.97	1.47	0.12	25.20	− 1.36	0.18	0.71	0.14	27.64	0.63	0.53	1.45	0.13	24.70	0.99	0.32	1.51	0.10	24.70
Left cingulum	0.71	0.48	2.73	< 0.001	25.34	0.50	0.61	0.95	0.81	26.50	− 0.02	0.98	1.15	0.58	27.42	1.12	0.27	1.47	0.12	25.81
Right superior fronto-occipital fasciculus	0.70	0.48	1.57	0.07	26.50	1.88	0.06	1.35	0.22	24.98	− 0.22	0.83	2.51	< 0.001	27.55	2.14	0.03	2.37	< 0.001	25.14
Left superior fronto-occipital fasciculus	0.85	0.40	1.66	0.04	27.02	2.10	0.04	1.53	0.09	23.90	2.16	0.03	3.61	< 0.001	26.12	1.05	0.29	1.04	0.88	25.71
Right sagittal stratum	1.29	0.20	1.23	0.40	26.85	1.34	0.18	1.93	0.01	27.71	− 0.72	0.47	0.88	0.57	28.30	1.85	0.07	2.40	< 0.001	26.80
Left sagittal stratum	2.03	0.04	1.25	0.36	24.74	2.48	0.01	2.28	< 0.001	25.98	− 1.07	0.29	0.66	0.07	27.39	2.63	0.01	1.76	0.02	24.41

### 3.3 Associations with sample characteristics

**Fig. 7:**
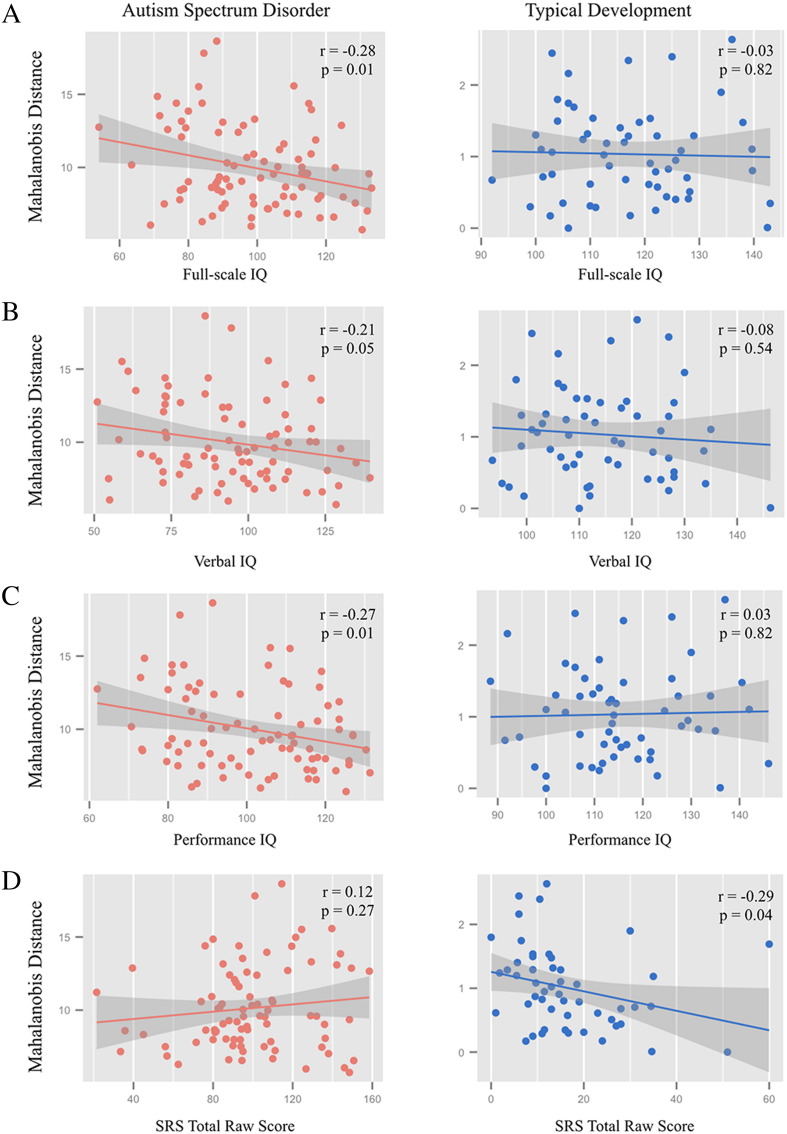
Associations of Mahalanobis distance with phenotypic characteristics. Scatter plots of the relationships between D_M_ and full-scale IQ (A), verbal IQ (B), performance IQ (C), and SRS total raw scores (D). Correlations within the ASD group (*left panel*) were found to be significant between D_M_ and full-scale, verbal and performance IQ. Correlations with IQ were not observed to be significant in the TDC participants (*right panel*). In the ASD group, a positive trend between SRS total raw and D_M_ was observed, however this association was not significant. Please note the ordinate axis scale differences between ASD and TDC scatter plots.

To investigate whether microstructural differences corresponded to phenotypic characteristics of the sample, correlations between D_M_ and full-scale IQ, verbal IQ, performance IQ and the Social Responsiveness Scale total raw score were computed. Scatter plots depicting these correlations are shown in Fig. 7. Correlations between D_M_ and IQ characteristics were not observed to be significant in control participants, however, a negative relationship between SRS total raw score and D_M_ was observed (r = − 0.29, p = 0.04). In autism participants, increased D_M_ was significantly associated with lower full-scale IQ (r = − 0.28, p = 0.01), lower performance IQ (r = − 0.27, p = 0.01), and lower verbal IQ (r = − 0.21, p = 0.05). SRS total raw score showed a positive relationship with D_M_ (increased autism severity were associated with increased deviation from normal); however, this also did not reach statistical significance (r = 0.12, p = 0.27).

## 4 Discussion

In this work, we have used the Mahalanobis distance (D_M_) as a metric to characterize complex and subtle relationships of multivariate measures of individual brain microstructure within a longitudinal sample of individuals with autism spectrum disorder. We show that D_M_ formed from DTI measures of FA, MD, AD, and RD across regions of white matter provides the greatest separation between ASD and TDC groups compared to univariate measures and to D_M_ computed from these parameters separately. These results substantiate that ASD impacts not only a single brain region or white matter tract, but rather that ASD affects multiple brain regions, re-confirming that it is a heterogeneous neurodevelopmental disorder. Furthermore, our findings suggest that D_M_ may be effective at characterizing the degree of brain differences within individuals with ASD as well as informing the microstructural heterogeneity and phenotypic characteristics of individuals.

Evaluation of the degree to which an individual deviates from a normative reference is informative for assessing brain differences and shifting from analyses focused on group-based differences to measures aimed at characterizing individual differences. Standardized scores of head circumference, brain volume, and other brain features have been routinely used to assess the distribution of brain characteristics in individuals with ASD (Barnea-Goraly et al., 2004, Courchesne et al., 2003, Herbert et al., 2004, Lainhart et al., 2006, Lainhart et al., 1997, Miles et al., 2000, Prigge et al., 2013), as well as other disorders, including mild traumatic brain injury (Kim et al., 2013, Lipton et al., 2012), bipolar disorder (Johnson et al., 2015), multiple sclerosis (Poonawalla et al., 2010), and Alzheimer's disease (Matsuda, 2014), among others. However, as it becomes commonplace to acquire multi-modal information from study participants, it becomes necessary to account for the multidimensional aspects of data. D_M_ provides a direct multivariate extension of a common univariate score, while, importantly, accounting for the variance of each variable and covariance between variables. This is essential given that neuroimaging measures can have different scales and be inherently correlated within and between different imaging modalities (Hao et al., 2013, Kulikova et al., 2014). Hence, while the current study has presented D_M_ in ASD, this measure may be informative to assess individual brain differences in a broader range of disorders, including mild traumatic brain injury, bipolar disorder, and Alzheimer's disease.

To our knowledge, the current study is the first to use the Mahalanobis distance to evaluate brain variation in ASD. Recently, Kulikova et al., (2014) used D_M_ to combine complementary measures of white matter to examine the brain development in healthy infants and found that D_M_ captured maturational relationships more reliably than univariate measures (Kulikova et al., 2014). D_M_ has additionally been used to differentiate individuals with Alzheimer's disease and mild cognitive impairment (Lindemer et al., 2015) and distinguish individuals with schizophrenia from healthy controls (Caprihan et al., 2008). Similarly, our findings suggest that multivariate approaches may be advantageous for describing individual brain deviations in ASD compared to univariate techniques. These results are consistent with other recent efforts that have utilized alternative multivariate analysis strategies in the study of ASD (for a review, see (Levman and Takahashi, 2015)). For example, support vector machines and other machine learning strategies have demonstrated the ability to accurately distinguish individuals with ASD from typically developing controls using a variety of neuroimaging measures as well as identify salient brain regions and networks that are implicated in core behavioral and social deficits (Ecker et al., 2010, Jin et al., 2015, Just et al., 2014, Lange et al., 2010, Uddin et al., 2011, Wee et al., 2014). Taken together, this corpus of research suggests that these analysis strategies improve our ability disentangle complementary information provided by multivariate brain measures and thus better characterize the anatomical, functional, and microstructural heterogeneity of ASD. This is essential for being able to identify possible ASD subgroups or specific individuals with unique brain features that are significantly different from both unaffected controls and peers with ASD.

Neuroimaging studies have provided great insight into the neurobiological changes that occur in ASD and have provided extensive evidence of widespread brain alterations that take place over the lifespan (for a review see Ameis and Catani, 2015, Ecker et al., 2015, Travers et al., 2012). However, while many of these studies have highlighted the complexity of these brain differences, where numerous regions and networks across the brain are involved, these brain regions and networks have typically been studied separately. Such approaches are instructive to understand the role of each particular brain region or network; however, the diversity of neuroimaging findings continues to suggest that a specific disparity may not be experienced by all individuals with ASD. Indeed, in comparing white matter regions between ASD and TDC groups, we observed differences across white matter in both the group mean and variance of the standard score distributions of DTI parameters (i.e. FA, MD, AD, and RD), particularly in the corpus callosum (genu and body), superior longitudinal fasciculi, and superior fronto-occipital fasciculi. Nevertheless, by combining regional measures of white matter microstructure across the brain to form D_M_, we are able to leverage the multivariate information from each of these brain regions to calculate a measure that describes the degree to which these collective brain measures differ from a normative reference.

In addition to D_M_ providing a measure of brain deviation, examination of the distributions of D_M_ (Fig. 3, Fig. 4, Fig. 5) and regional standardized scores (Fig. 6) across individuals provided insights into the microstructural heterogeneity of ASD. In particular, the variance of D_M_ distributions were found to be significantly increased, while this increased variation was observed in only a small proportion of univariate distributions. Increased variation has similarly been reported throughout studies of autism (Alexander et al., 2007, Lainhart, 2006, Lange et al., 2010, Miles et al., 2000, Prigge et al., 2013, Travers et al., 2015b), which suggests brain alterations do not necessarily impact specific brain regions in the same way, but rather have a varied impact across individuals with ASD. The use of multivariate techniques is, therefore, advantageous over other approaches, such as the use of signal histograms and univariate distributions, as they enable a broader characterization of underlying alterations. Furthermore, unlike signal histograms, D_M_ can incorporate multiple image contrasts that provide an additional dimension of capturing brain alteration. The ability to identify and characterize the heterogeneous alterations observed in ASD, as presented in the current study, is thus an important aspect for both identifying and understanding specific neurobiological mechanisms of autism.

For individuals with autism, we found that increased D_M_ (i.e. increased brain deviation) was associated with decreased full-scale, performance, and verbal IQ's. However, while the relationship between SRS total raw scores and D_M_ was positive, the correlations were not observed to be significant. Consistent with our results, other studies have found connections between microstructural neuroimaging indices and phenotypic and symptomology measures (Alexander et al., 2007, Noriuchi et al., 2010, Travers et al., 2015a). These associations are particularly informative in identifying neural mechanisms as these measures are consistently used to assess core diagnostic and phenotypic characteristics in autism. In all, this collective body of research indicates a link between underlying white matter microstructure and ASD symptomology and severity.

Despite our findings suggesting widespread white matter microstructural differences in ASD, D_M_ does not provide information into which specific microstructural feature(s) drives the observed individual difference. For example, one individual may be observed to have an abnormal corpus callosum microstructure while having a normal appearing anterior internal capsule. Another individual may have a deviant anterior internal capsule microstructure, but appear to have a normative corpus callosum. Even still, another individual may have a differential developmental trajectory in both of these regions. Yet, the D_M_ for each of these cases could be identical. As observed in the current study, including multiple DTI parameters may increase the separability of the ASD and TDC distributions, which suggests that each of these measures uniquely contribute to D_M_. However, D_M_ provides an omnibus measure of individual brain difference and therefore interpretation of the specific microstructural characteristics or processes resulting in the observed difference, such as a difference in the developmental trajectory, is limited. The use of decomposition methods, such as principal component analysis (PCA), could be used in combination with D_M_ to identify the underlying brain feature(s) that are subsequently altered. Furthermore, while measures derived from DTI (i.e. FA and MD) are sensitive to underlying changes of white matter, these parameters lack specificity to underlying microstructural changes (Alexander et al., 2011, Beaulieu, 2002, Jones et al., 2013). Incorporating additional measures, such as head circumference (Courchesne et al., 2003, Lainhart et al., 2006, Lainhart et al., 1997), volumetric measurements (Bigler et al., 2010, McAlonan et al., 2005), indices of cortical thickness (Zielinski et al., 2014) and functional connectivity (Anderson et al., 2011, Cherkassky et al., 2006) as well as other white matter markers (Alexander et al., 2011, Dean et al., 2016, Deoni et al., 2014, Zhang et al., 2012) will likely alter the covariance between parameters and alter the values of D_M_. This may help to improve the interpretation of brain differences observed in ASD as well as provide further improvements in discriminating between ASD and TDC individuals.

In addition to understanding the macrostructural and microstructural characteristics of the brain that may be disrupted in ASD, converging evidence points to altered brain development as having a fundamental role (Courchesne, 2004, Lainhart, 2003, Lange et al., 2015, Lewis and Elman, 2008, Travers et al., 2015b, Wolff et al., 2012). The human brain undergoes great changes over the course of the lifespan (Casey et al., 2005, Croteau-Chonka et al., 2016, Dean et al., 2014b, Deoni et al., 2012, Evans and Brain Development Cooperative Group, 2006, Giedd et al., 1999, Giedd and Rapoport, 2010, Lange et al., 1997, Lebel and Beaulieu, 2011), while such developmental processes have a critical role in establishing both structural and functional brain networks that ultimately enable the processing of complex information (Durston and Casey, 2006). Here, generalized additive mixed effects models were used to characterize longitudinal developmental trajectories of the examined white matter regions, allowing us to account for nonlinear neurodevelopmental changes across our sample and enabling a direct comparison of groups and individuals of all ages. However, while the variability of brain imaging measures beyond the average growth trajectory may change with age, individual brain deviations at one age may differ at another age. While in the current study we averaged across the longitudinal time points to establish an overall individual measure of deviation, future studies may examine the changes of D_M_ with age. Indeed, D_M_ has been used to depict the development of white matter in healthy infants (Kulikova et al., 2014), and thus such future studies investigating the age-relationships of D_M_ in ASD may provide important insights about the timing of abnormal brain deviations in ASD and help identify when such brain changes first begin to appear.

While the presented study provides strong evidence of D_M_ as an informative measure of individual brain deviation, there are several limitations to this study. First, the formation D_M_ relies on the assumption that the TDC growth trajectory can be used as a normative reference for the examined sample. Although typical development was confirmed using extensive neuropsychological assessments and detailed medical history information, it is possible that some participants may later develop atypically and thus not be representative of a normal population. Similarly, we presumed that the smoothed splines of the generalized additive mixed effects models used herein provided a characteristic representation of the typically developing growth trajectory. While these models have been shown to effectively model growth trajectories in the corpus callosum (Travers et al., 2015b), additional models have been used to characterize the developmental trajectories of DTI (Lebel and Beaulieu, 2011, Sadeghi et al., 2013) and other imaging parameters (Dean et al., 2014a, Shaw et al., 2008, Zielinski et al., 2014). Future studies comparing growth models that describe the neurodevelopmental trajectories are therefore needed to determine the best current models of brain development in ASD. Lastly, D_M_ provides limited interpretability beyond being able to characterize the magnitude of deviation. For example, it may be informative to identify whether certain ASD brain regions are abnormally enlarged or reduced compared to that of typically developing individuals. Incorporating measures of D_M_ with multivariate classification techniques (Ecker et al., 2010, Lange et al., 2010, Uddin et al., 2011) may help determine the relative weighting that each brain region contributes to the overall distance measure.

## 5 Conclusion

Heterogeneity of brain-based descriptors is a fundamental feature of ASD that makes identification of neuroimaging-based phenotypes challenging. However, multivariate measures, such as the Mahalanobis distance, are able to incorporate this inherent heterogeneity and provide an informative descriptor about an individual's overall deviation from a representative reference sample. In particular, the results from the present study suggest that the Mahalanobis distance formed from multiple measures of white matter microstructure can provide an increased degree of separation between individuals with and without autism, compared to more commonly used univariate approaches. In particular, these results suggest that the Mahalanobis distance of brain features may provide a novel measure that may be informative to identify autism sub-groups or severely-impaired individuals with autism. This has particular value for future studies evaluating genetic and environmental risk factors that are associated with specific neurobiological mechanisms of autism.

The following are the supplementary data related to this article.

## Funding

This work was supported by the National Institute of Mental Health [RO1 MH080826 to JEL, ALA, NL, EDB; RO1 MH084795 to JEL, PTF, NL; RO1 MH097464 to JEL, ML, NL, ALA; K08 MH100609 to BAZ, and KO8 MH092697 to JSA], the Eunice Kennedy Shriver National Institute of Child Health and Human Development [T32 HD007489 to DCD, BGT, and P30 HD003352 to the Waisman Center], the Poelman Foundation [to EDB], the Primary Children's Foundation [Early Career Development Award to BAZ], Brain and Behavior Research Foundation [NARSAD Young Investigator Award to BGT], the Hartwell Foundation [BGT]. The content is solely the responsibility of the authors and does not necessarily represent the official views of the National Institute of Mental Health, the National Institute of Child Health & Development, or the National Institutes of Health.
